# Three-Dimensional Reconstruction of Thoracic Structures: Based on Chinese Visible Human

**DOI:** 10.1155/2013/795650

**Published:** 2013-11-24

**Authors:** Yi Wu, Na Luo, Liwen Tan, Binji Fang, Ying Li, Bing Xie, Kaijun Liu, Chun Chu, Min Li, Shaoxiang Zhang

**Affiliations:** ^1^Institute of Computing Medicine and Department of Human Anatomy, Third Military Medical University, Chongqing 400038, China; ^2^Department of Dermatology, Southwest Hospital, Third Military Medical University, Chongqing 400038, China; ^3^Institute of Bioengineering, Chongqing University, Chongqing 400044, China

## Abstract

We managed to establish three-dimensional digitized visible model of human thoracic structures and to provide morphological data for imaging diagnosis and thoracic and cardiovascular surgery. With Photoshop software, the contour line of lungs and mediastinal structures including heart, aorta and its ramus, azygos vein, superior vena cava, inferior vena cava, thymus, esophagus, diaphragm, phrenic nerve, vagus nerve, sympathetic trunk, thoracic vertebrae, sternum, thoracic duct, and so forth were segmented from the Chinese Visible Human (CVH)-1 data set. The contour data set of segmented thoracic structures was imported to Amira software and 3D thorax models were reconstructed via surface rendering and volume rendering. With Amira software, surface rendering reconstructed model of thoracic organs and its volume rendering reconstructed model were 3D reconstructed and can be displayed together clearly and accurately. It provides a learning tool of interpreting human thoracic anatomy and virtual thoracic and cardiovascular surgery for medical students and junior surgeons.

## 1. Introduction

The thorax is an important chamber of human body and is protected by the thoracic wall including thoracic cage and associated skin, muscle, and fascia. It is separated from the abdominal cavity by the diaphragm and the upper limit is formed by the manubrium in front, the first ribs laterally, and the spine posteriorly. It contains the main organs relative to respiration and circulation including lung, trachea, bronchia, heart, aorta, superior vena cava, inferior vena cava, and so forth, which nearly control human life. The thoracic diseases including lung cancer, thymus neoplasm, cardiac surgery disease, aortic aneurysm, and mediastinal tumor of nervous tissue are prevalent. If the cardiovascular and thoracic surgeons cannot master the anatomy of human thoracic structures, they may injure thoracic structures such as pulmonary lobe, pericardium, aorta, esophagus, trachea, bronchia, and vagus nerve during operation, leading to pneumothorax, hematorrhea, vagus nerve injury, or many other unwanted sever consequences. Previous anatomical data of lung and mediastinum including clinical image examinations are mostly two-dimensional (2D) images such as CT, CTA, MRI, MRA, and anatomical atlas, obviously, but 2D data cannot fully and faithfully reveal the three-dimensional (3D), full-scale information of the thoracic organs, and sometimes ambiguous conclusions may be reached. This kind of situation cannot efficiently meet the clinical requirement [1–4].

In recent years, theoretical and applied anatomical studies have been carried out on the basis of the American Visible Human Project (VHP) data sets, and visible human models have also been playing an important role in surgical teaching and clinical practice [5–11]. At present, there are several data sets of adult Chinese men and women in China, which lay the foundation for the research on the visible thoracic structures of Chinese people; so far the report of detailed 3D visible thoracic structures' model including lung and mediastinal structures is still not described. So in our study, a 3D visible model of the thoracic structure was established using CVH-1 data set. The study aims to help medical students and surgeons to master the complicated thoracic structures and to provide a basis for building dynamic thoracic structure and performing virtual cardiovascular and thoracic operations.

## 2. Materials and Methods

### 2.1. Retrieval of Sectional Anatomical Images of Thoracic Structures

The serial cross-sectional images of thoracic structures were prepared by using the first Chinese visible human (CVH-1) data sets. The cadaver of CVH-1 is a middle-aged, middle-sized, well-developed cadaver (sex: male; age: 35 years old; body height: 1,700 mm; body weight: 65.0 kg) without organic lesions and has been enrolled into the cadaver donation program. Both donor and his relatives will donate his body to the visible human program, which follows scientific ethic rules of Third Military Medical University and Chinese Ethics Department. 

Successive cross-sectional images from the neck (1400) to the upper abdomen (1700) were retrieved from CVH-1 data set. The slice was 1.0 mm thick, and the cross-sections were photographed by digital camera (Canon, made in Japan) at a high-resolution of 6,291, 456 (3,072 × 2,048) pixels [1]. Every cross-section can successively and fully reveal the thoracic organs such as lung, heart, aorta, and so forth. There are 301 slices which we used in our study.

### 2.2. Segmentation on 2D Images

**Figure 1:**
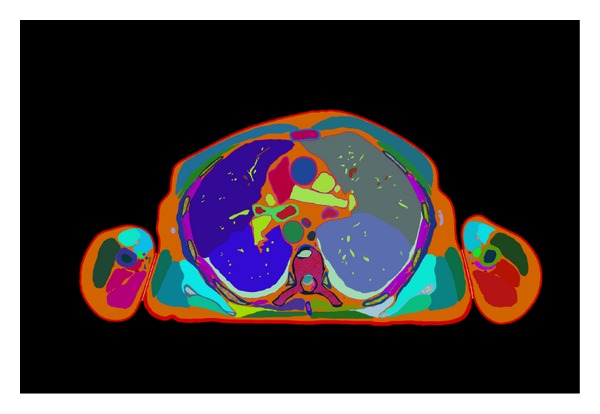
Segmentative image of CVH thorax.

After registration through four reserved fiducial rods, segmentation was used to outline the thoracic structures on the 2D images with Photoshop 8.0 software (interface is shown in Figure 1). During segmentation, lung, heart, aorta and its ramus, trachea, bronchia, azygos vein, superior vena cava, inferior vena cava, thymus, esophagus, diaphragm, phrenic nerve, vagus nerve, sympathetic trunk, thoracic vertebrae, sternum, and thoracic duct were outlined on 2D sections with magnetic lasso tool. Then each structure was established as a layer and was filled with different RGB colors. Each image layer of segmental anatomical structure was assigned an anatomical name and the files were saved as PSD format. As to some structures such as phrenic nerve, vagus nerve, and sympathetic trunk which were difficult to distinguish where nervous tissue and the adjacent connective tissue mix, contour lines were extracted in some slices where nervous tissue can be observed.

### 2.3. Three-Dimensional Reconstruction and Visualization

**Figure 2:**
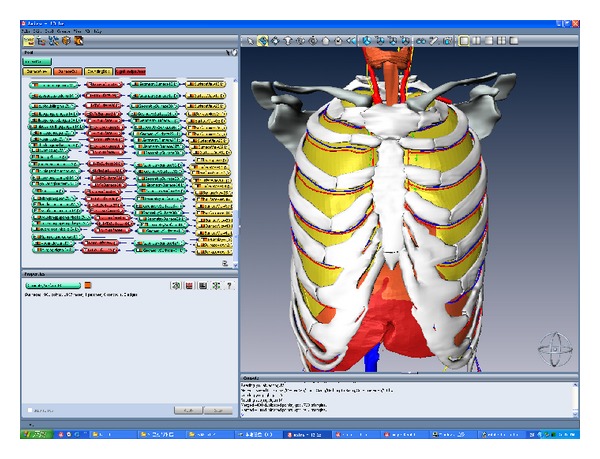
Amira software's interface.

After segmentation, the segmented structures were surface rendering reconstructed with Amira 5.2 software (interface is shown in Figure 2). Surface rendering is outlining the contour of the anatomical structures from 2D images and forming 3D surface with triangle grid. As to nervous tissue, interpolation was used in some slices where the nervous tissue cannot be observed clearly, which can be helpful to form consecutive tissue structures. The 3D reconstructed model of nervous tissue was imported to MAYA software and 3D reconstructed to smooth NURBS model.

### 2.4. Combining Surface Rendering and Volume Rendering Reconstruction

Preserving 3D surface rendering reconstructed model of thoracic organs, above 301 serial transverse cross-sectional images of thoracic structures from CVH-1 data set were imported to Amira software and three-dimensional reconstructed via volume rendering. The 3D reconstructed surface and volume rendering model of thoracic structures were observed clearly and directly using the orthoslice and oblique slice features of the software.

## 3. Results

### 3.1. A Three-Dimensional Model Established by Using Amira Software

The thorax model including lung, heart, aorta and its ramus, azygos vein, superior vena cava, inferior vena cava, trachea, bronchia, thymus, esophagus, diaphragm, phrenic nerve, vagus nerve, sympathetic trunk, thoracic vertebrae, sternum, and thoracic duct, which can represent Asia population, was 3D surface-rendering and volume-rendering reconstructed by using Amira software and presented no data loss. The resolution of the cross-section images reached up to 6.3 × 10^6^ pixels. The serial digital images allowed a unique anatomical insight into the thoracic cavity, showing preferably the subtle anatomical structures of thorax. The three-dimensional model can be displayed partially or as a whole or with some structures being transparent and can be observed from any orientation, angle, or position of view. The reconstructed three-dimensional images can demonstrate two-dimensional sectional anatomical structure, which can provide the correct anatomical information, measure the volume and surface area of any reconstructed structure, and also simulate thoracic anatomy from any plane.

**Figure 3:**
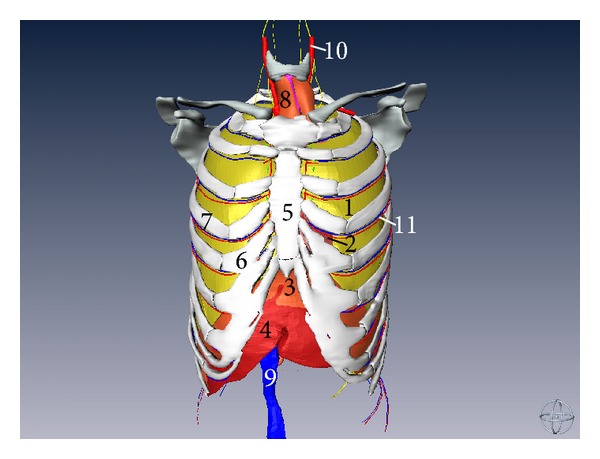
Three-dimensional surface rendering reconstructed model of thoracic structures with thoracic cage (anterior aspect) (1) left lung, (2) heart, (3) diaphragm, (4) liver, (5) sternum, (6) costal cartilage, (7) costal bone, (8) thymus, (9) inferior vena cava, (10) common carotid artery, and (11) intercostal artery and vein.

**Figure 4:**
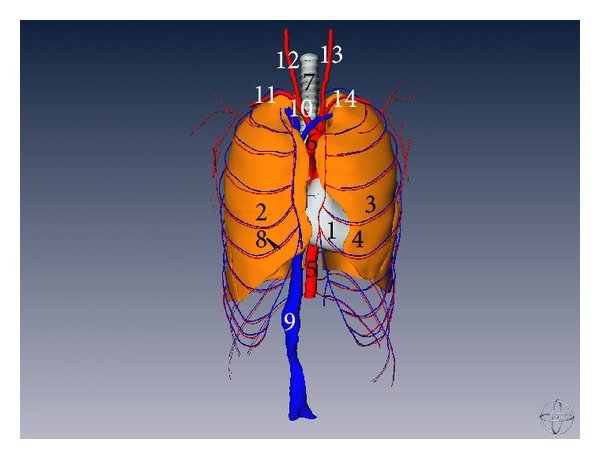
Three-dimensional reconstructed model of thoracic structures without thoracic cage (anterior aspect) (1) heart, (2) right lung, (3) left lung, (4) lingula of left lung, (5) abdominal aorta, (6) aorta arch, (7) trachea, (8) intercostal artery and vein, (9) inferior vena cava, (10) brachiocephalic trunk, (11) right subclavian artery, (12) right common carotid artery, (13) left common carotid artery, and (14) left subclavian artery.

Two lungs are nearly embedded by bony thoracic cage. The apex of both lungs is a little above the clavicle and the base of lung is concave. The lingual of left lung is in front of the left border of the heart. The heart is located in the middle mediastinum of the thorax and rest upon the diaphragm. Nearly two-third of the heart lies on the left of the median sagittal plane and the whole heart is nearly embedded by the thoracic cage but that only some part of the heart can show in the intercostal space (as shown in Figures 3 and 4).

**Figure 5:**
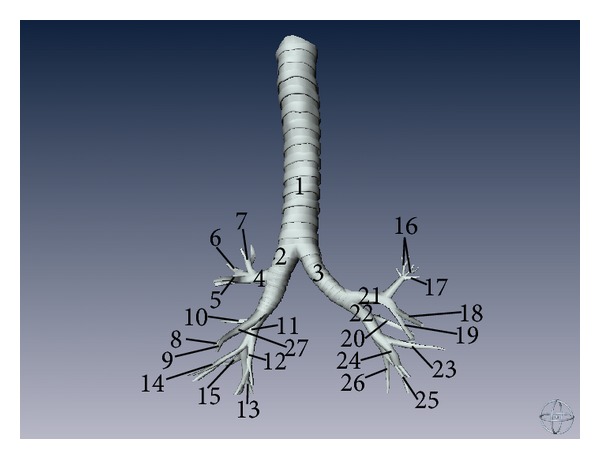
Three-dimensional model of trachea and bronchi (anterior aspect). (1) Trachea, (2) left bronchi, (3) right bronchi, (4) right superior lobar bronchus, (5) anterior segmental bronchus (BIII) (right lung), (6) posterior segmental bronchus (BII) (right lung), (7) apical segmental bronchus (BI) (right lung), (8) lateral segmental bronchus (BIV) (right lung), (9) medial segmental bronchus (BV) (right lung), (10) superior segmental bronchus (BVI) (right lung), (11) right inferior lobar bronchus, (12) medial basal segmental bronchus (BVII) (right lung), (13) posterior basal segmental bronchus (BX) (right lung), (14) anterior basal segmental bronchus (BVIII) (right lung), (15) lateral basal segmental bronchus (BIX) (right lung), (16) apicoposterior segmental bronchus (BI+BII) (left lung), (17) anterior segmental bronchus (BIII) (left lung), (18) superior lingular bronchus (BIV) (left lung), (19) inferior lingular bronchus (BV) (left lung), (20) superior segmental bronchus (BVI) (left lung), (21) left superior lobar bronchus, (22) left inferior lobar bronchus, (23) anterior basal segmental bronchus (BVIII) (left lung), (24) medial basal segmental bronchus (BVII) (left lung), (25) lateral basal segmental bronchus (BIX) (left lung), (26) posterior basal segmental bronchus (BX) (left lung), and (27) right middle lobar bronchus.

**Figure 6:**
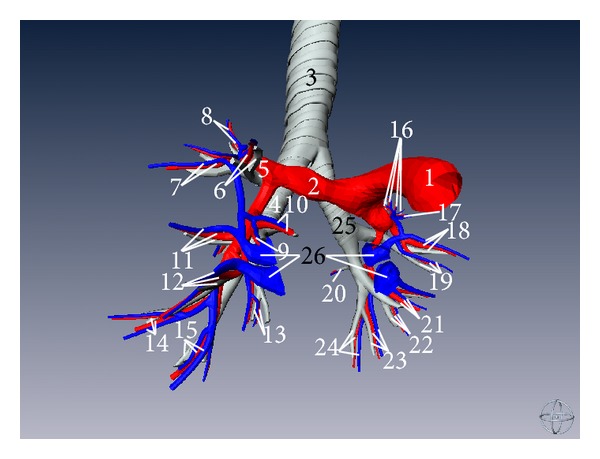
Three-dimensional model of trachea, bronchial tree, pulmonary artery and its ramus, and pulmonary vein and its ramus (anterior aspect). (1) Pulmonary artery, (2) right pulmonary artery, (3) trachea, (4) right bronchi, (5) superior lobar branches of right pulmonary artery, (6) anterior segmental bronchus (BIII) and its corresponding branch of pulmonary artery and vein (right lung), (7) posterior segmental bronchus (BII) and its corresponding branch of pulmonary artery and vein (right lung), (8) apical segmental bronchus (BI) and its corresponding branch of pulmonary artery and vein (right lung), (9) lateral segmental bronchus (BIV) and its corresponding branch of pulmonary artery and vein (right lung), (10) medial segmental bronchus (BV) and its corresponding branch of pulmonary artery and vein (right lung), (11) superior segmental bronchus (BVI) and its corresponding branch of pulmonary artery and vein (right lung), (12) anterior basal segmental bronchus (BVIII) and its corresponding branch of pulmonary artery and vein (right lung), (13) medial basal segmental bronchus (BVII) and its corresponding branch of pulmonary artery and vein (right lung), (14) lateral basal segmental bronchus (BIX) and its corresponding branch of pulmonary artery and vein (right lung), (15) posterior basal segmental bronchus (BX) and its corresponding branch of pulmonary artery and vein (right lung), (16) apicoposterior segmental bronchus (BI + BII) and its corresponding branch of pulmonary artery and vein (left lung), (17) anterior segmental bronchus (BIII) and its corresponding branch of pulmonary artery and vein (left lung), (18) superior lingular bronchus (BIV) and its corresponding branch of pulmonary artery and vein (left lung), (19) inferior lingular bronchus (BV) and its corresponding branch of pulmonary artery and vein (left lung), (20) superior segmental bronchus (BVI) and its corresponding branch of pulmonary artery and vein (left lung), (21) anterior basal segmental bronchus (BVIII) and its corresponding branch of pulmonary artery and vein (left lung), (22) medial basal segmental bronchus (BVII) and its corresponding branch of pulmonary artery and vein (left lung), (23) lateral basal segmental bronchus (BIX) and its corresponding branch of pulmonary artery and vein (left lung), and (24) posterior basal segmental bronchus (BX) and its corresponding branch of pulmonary artery and vein (left lung).

All segmental bronchi of two lungs are 3D reconstructed and detailed relationship of lobar bronchi and segmental bronchi can be observed and the ramus of pulmonary artery and vein accompany corresponding bronchi (as shown in Figures 5 and 6).

**Figure 7:**
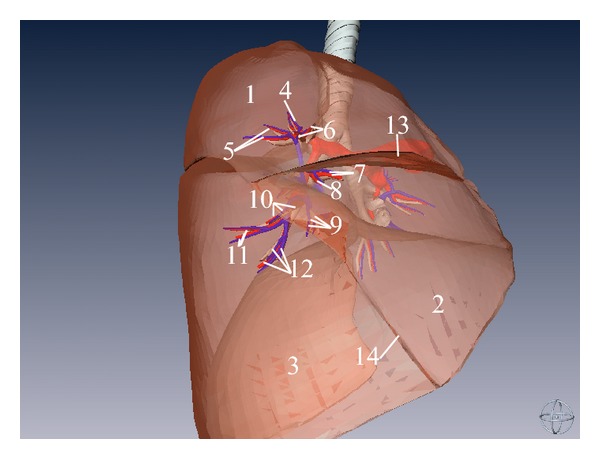
Three-dimensional model of right lung and its corresponding bronchial tree, pulmonary artery, pulmonary vein (right aspect). (1) Superior lobe of right lung, (2) middle lobe of right lung, (3) inferior lobe of right lung, (4) apical segmental bronchus (BI) and its corresponding branch of pulmonary artery and vein (right lung), (5) posterior segmental bronchus (BII) and its corresponding branch of pulmonary artery and vein (right lung), (6) anterior segmental bronchus (BIII) and its corresponding branch of pulmonary artery and vein (right lung), (7) medial segmental bronchus (BV) and its corresponding branch of pulmonary artery and vein (right lung), (8) lateral segmental bronchus (BIV) and its corresponding branch of pulmonary artery and vein (right lung), (9) medial basal segmental bronchus (BVII) and its corresponding branch of pulmonary artery and vein (right lung), (10) anterior basal segmental bronchus (BVIII) and its corresponding branch of pulmonary artery and vein (right lung), (11) lateral basal segmental bronchus (BIX) and its corresponding branch of pulmonary artery and vein (right lung), (12) posterior basal segmental bronchus (BX) and its corresponding branch of pulmonary artery and vein (right lung), (13) horizontal fissure of right lung, and (14) oblique fissure.

Right lung is divided into superior, middle, and inferior lobe by a horizontal fissure and an oblique fissure of right lung. Apical, posterior, and anterior segmental bronchus (BI + BII + BIII) enter superior lobe, and lateral and medial segmental bronchus (BIV + BV) enter middle lobe, superior (it cannot be observed clearly because of organs wrapping), medial basal, anterior basal, lateral basal, and posterior basal segmental bronchus (BVI + BVII + BVIII + BIX + BX) enter inferior lobe (As shown in Figure 7).

**Figure 8:**
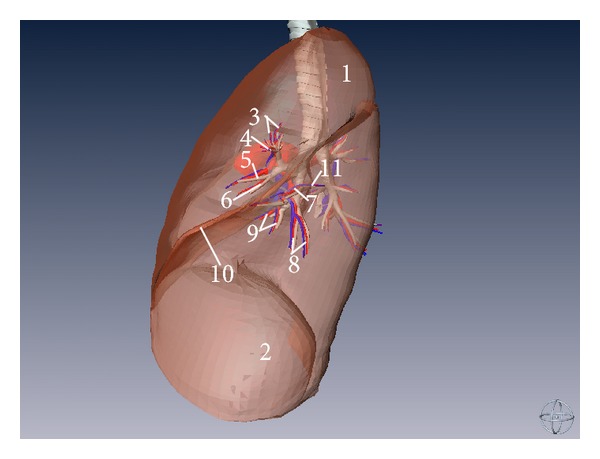
Three-dimensional model of left lung and its corresponding bronchial tree, pulmonary artery, and pulmonary vein (left aspect). (1) Superior lobe of left lung, (2) inferior lobe of left lung, (3) apicoposterior segmental bronchus (BI + BII) and its corresponding branch of pulmonary artery and vein (left lung), (4) anterior segmental bronchus (BIII) and its corresponding branch of pulmonary artery and vein (left lung), (5) superior lingular bronchus (BIV) and its corresponding branch of pulmonary artery and vein (left lung), (6) inferior lingular bronchus (BV) and its corresponding branch of pulmonary artery and vein (left lung), (7) anterior basal segmental bronchus (BVIII) and its corresponding branch of pulmonary artery and vein (left lung), (8) lateral basal segmental bronchus (BIX) and its corresponding branch of pulmonary artery and vein (left lung), (9) posterior basal segmental bronchus (BX) and its corresponding branch of pulmonary artery and vein (left lung), (10) oblique fissure, and (11) superior segmental bronchus (BVI) and its corresponding branch of pulmonary artery and vein (left lung).

Left lung is divided into superior and inferior lobe by an oblique fissure of left lung. Apical, posterior, anterior, superior lingual, and inferior lingual segmental bronchus (BI+BII+BIII +BIV+BV) enter superior lobe, and superior (it cannot be observed clearly because of organs wrapping), medial basal, anterior basal, lateral basal, and posterior basal segmental bronchus (BVI + BVII + BVIII + BIX + BX) enter inferior lobe (As shown in Figure 8).

**Figure 9:**
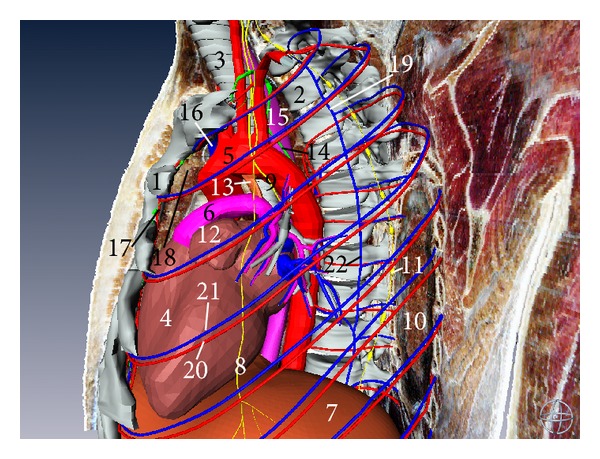
Three-dimensional model of mediastinal organs (left aspect). (1) Sternum, (2) thoracic vertebrae, (3) trachea, (4) heart, (5) aorta, (6) pulmonary artery, (7) diaphragm, (8) phrenic nerve, (9) vagus nerve, (10) left lung, (11) sympathetic trunk, (12) left auricle of the heart, (13) arterial ligament, (14) thoracic duct, (15) esophagus, (16) superior vena cava, (17) parasternal lymph nodes, (18) thymus, (19) accessory hemiazygos vein, (20) posterior intercostal artery, (21) posterior intercostal vein, and (22) hemiazygos vein.

After left lung is hidden, the apex of the heart, left auricle of the heart, and the root of left lung can be seen. Among the triangle of ductus arteriosus, which is made up of left vagus nerve, left phrenic nerve, and superior border of pulmonary artery, arterial ligament can be observed clearly. Thymus is located in anterior mediastinum, which is between sternum and pericardium. Esophagus is right and anterior to thoracic aorta. Sympathetic trunk is in front of and close to the transverse process of the thoracic vertebra. Hemiazygos and accessory hemiazygos are located in the anterior and left surface of the thoracic vertebrae and receive many posterior intercostal veins. Left vagus nerve descends between left common carotid and left subclavian arteries, crosses in front of the aortic arch, passes behind the root of left lung, and reaches the anterior side of esophagus (as shown in Figure 9).

**Figure 10:**
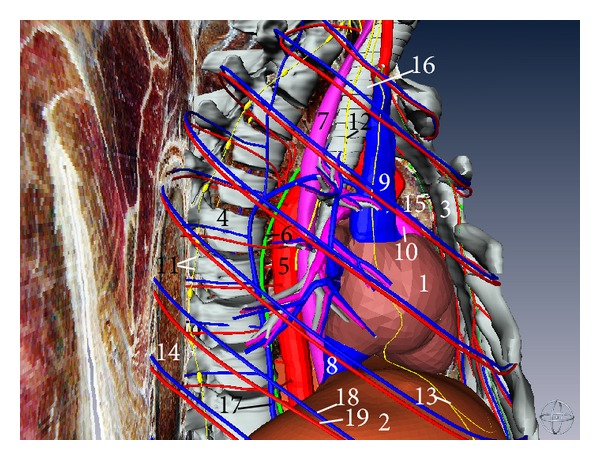
Three-dimensional model of mediastinal organs (right aspect). (1) Heart, (2) diaphragm, (3) sternum, (4) thoracic vertebrae, (5) thoracic aorta, (6) thoracic duct, (7) esophagus, (8) inferior vena cava, (9) superior vena cava, (10) pulmonary artery, (11) sympathetic trunk, (12) right vagus nerve, (13) phrenic nerve, (14) right lung, (15) thymus, (16) trachea, (17) azygos vein, (18) posterior intercostal vein, and (19) posterior intercostal artery.

**Figure 11:**
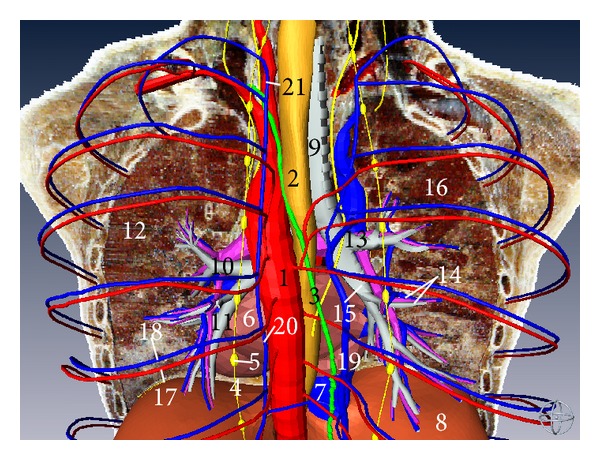
Three-dimensional model of mediastinal organs (posterior aspect). (1) Thoracic aorta, (2) esophagus, (3) thoracic duct, (4) sympathetic trunk, (5) ganglia of sympathetic trunk, (6) heart, (7) inferior vena cava, (8) diaphragm, (9) trachea, (10) left superior lobar bronchus, (11) left inferior lobar bronchus, (12) left lung, (13) right superior lobar bronchus, (14) right middle lobar bronchus and its corresponding ramus of pulmonary artery and vein, (15) right inferior lobar bronchus, (16) right lung, (17) posterior intercostal artery, (18) posterior intercostal vein, (19) azygos vein, (20) hemiazygos vein, and (21) accessory hemiazygos vein.

From the right view and posterior view of mediastinum, the heart, superior vena, inferior vena cava, and lung root of right lung can be observed clearly. At the upper part of thorax, esophagus is located between the trachea and thoracic vertebrae and deviates to the left. At the level of tracheal bifurcation, the esophagus lies posterior to trachea and anterior and left to the azygos vein. At the lower part of thorax (at the level of 8th to 9th thoracic vertebrae), the esophagus inclines to the left in front of the thoracic aorta. Thoracic duct ascends between the thoracic aorta and the azygos vein and then posterior to the esophagus. At the level of 4th thoracic vertebrae, it passes obliquely behind the esophagus to reach its left side. Right vagus nerve descends along the right side of the trachea, then passes behind the root of right lung, and reaches the posterior side of esophagus (as shown in Figures 10 and 11).

## 4. Discussion

### 4.1. Significance of CVH Male Data Set

Since visible three-dimensional reconstruction is based on two-dimensional images, the quality of original data provided by two-dimensional images is correlated to the fidelity of the reconstructed three-dimensional images. This CVH-1 data set can represent normal Asia-population thoracic anatomy and was more representative than the American VHP data set in terms of anatomical structures [12, 13]. Since most of structures in thorax are parenchyma, it is difficult to set up an archetypal male thorax model through CT, MRI, and ultrasound. However, on the high-resolution CVH 2D cross-sections, normal structures of the pericardium, bronchia, thymus, nervous tissue, and so forth can be displayed distinctly. Moreover, through artery perfusion with 20% red gelatin and the body frozen at −25°C, the CVH male project achieved greater integrity of images and easier blood vessel identification and were free of organic lesion (unlike the other visible human projects).

### 4.2. Three-Dimensional Reconstruction of Thorax

After registered and segmented with Photoshop 7.0 software, the sectional images of the data set of anatomical structures in thorax were 3D reconstructed via surface rendering based on marching cubes algorithm and volume rendering with Amira software. With surface rendering, structures can be freely visualized and displayed in three-dimensional space on personal computer partially or as a whole or with some of the structures being transparent. Surface rendering reconstruction needs only organic contour data, so it can save much disc space and can run faster than volume reconstruction. Using voxel as the basic modeling unit, volume rendering can show the rich internal details of object, which has become the preferred method of 3D medical visualization.

Volume rendering reconstruction can preserve the information of the original images and overcome the defects of the surface rendering (lacking internal anatomical information). The image of the volume rendering is of high quality, but the quantity of operation is too much and the computation cost of visualization is very high.

Combining surface rendering and volume rendering reconstruction, thoracic structures can be displayed clearly and truly. Comparing with surface rendering reconstruction or volume rendering reconstruction solely, this method can overcome above defects.

### 4.3. Significance of Establishment of Human Thorax Models

Many scientists 3D reconstructed tracheal tree, pulmonary artery, and pulmonary tree using CTA*∖*MRA images [14–18], while there is still no study reporting reconstruct of mediastinal parenchyma such as thymus, thoracic duct, lymph nodes, zygos vein, phrenic nerve, vagus nerve, and sympathetic nerve. Our 3D thorax model can provide detailed 3D reconstructed model of lung and mediastinal structures.

To familiarize and master the anatomy of lung and mediastinum and their relationship is critical to improve thoracic and cardiovascular surgical techniques and to increase the success rate of thoracic surgery. Mediastinum contains so many important structures such as heart, aorta and its ramus, azygos vein, superior vena cava, inferior vena cava, thymus, esophagus, diaphragm, phrenic nerve, vagus nerve, and sympathetic trunk, and the relationship of the thoracic structures is difficult and it is hard to touch. Prior to a thoracic surgery, combing with pathological information, surgeon can know the size and location of the thoracic lesion and its spatial relations to its surrounding structures, so as to design an individualized surgical plan. Otherwise, the accuracy of the plan of a thoracic surgery will be decreased greatly and it may lead to pneumothorax, hematorrhea, nerve, or thoracic duct injury. This three-dimensional reconstructed model of thoracic structures combining surface rendering with volume rendering can provide detailed and average information of Asian thorax. It can cut from any angle or any direction and display any sectional image including orthoslice and oblique slice.

Traditional anatomical study of thorax includes cadaver dissection and reading two-dimensional sectional images such as X-ray, CT, MRI, and anatomical atlas. In cadaver dissection, the anatomical structures and situation of some slim vessels and nerves in thorax are liable to be destroyed; moreover donated cadavers used in anatomical practice are scarce and limited. Two-dimensional images such as X-ray, CT, MRI, and anatomical atlas cannot 3D display the anatomical structures in the thorax, so medical students need more time to understand the anatomy of thorax. This 3D reconstructed anatomical model of thorax can provide detailed and integrated 3D anatomy in thorax for medical students learning gross anatomy and sectional anatomy [1, 2]. Detailed 3D anatomy may be observed from any direction or any angle. Medical students can dissect virtual thorax freely at any cut plane and can make any 3D reconstructed structures transparent, so it is helpful to observe its internal structure in a personal computer.

### 4.4. VR of Thorax

Virtual reality (VR) surgical simulation increasingly appears to be a promising aspect of the clinical anatomical education. The 3D reconstructed model of thoracic structures is a framework for simulating thoracic operation, thoracic radiotherapy, thoracoscopy and mediastinoscopy operation, and thoracic surgery rehearsal [19]. Junior surgeon can manipulate VR program of thorax to deeply master the detailed anatomy of thorax and thoracic surgical basic skills. For example, thoracoscopy and mediastinoscopy can be facilitated greatly by preclinical operation training by using a model-based simulation. In radical lobectomy or mediastinal tumour dissection, tumors should be identified and dissected, and adjacent normal structures are important and needed and should be carefully protected to some extent. Thoracic surgery rehearsal model based on this 3D reconstructed model will help thoracic and cardiovascular rehearse operation in virtual thorax, which is helpful to maximal tumor tissue dissection and to minimize operational trauma [20]. 

### 4.5. Some Problems

The 3D reconstructed models based on CVH data set can provide detailed thoracic anatomy and have a broad range of potential applications for modern medical diagnosis and therapy for thoracic diseases [21]. Nevertheless, lung and heart have their own movements, and the 3D geometric anatomic shapes are only initialized basement of constructing dynamic model of thorax and virtual reality. A huge amount of work still has to be done. The ultimate goal of our study is to produce a virtual dynamic photorealistic human model which has physical and even physiological function.
